# Supplementary material for: Impact of COVID-19 on Intracranial Meningioma Resection: Results from California State Inpatient Database
Source: Cancers (Basel). 2022 Sep 30;14(19):4785. doi: 10.3390/cancers14194785 (PMC9563583; doi:10.3390/cancers14194785)
Supplement: Supplementary file 1 [file cancers-14-04785-s001.zip › cancers-1836097-supplementary.pdf]

Supplementary Materials: Impact of COVID-19 on Intracranial Meningioma Resection: Results from California State Inpatient Database

Muni Rubens, Anshul Saxena, Venkataraghavan Ramamoorthy, Ashfaq Ahmed, Zhenwei Zhang, Peter McGranaghan, Emir Veledar and Michael McDermott

Table S1. International Classification of Diseases, Tenth Revision, Clinical Modification (ICD-10-CM) codes used for identifying Clavien-Dindo Grade IV complications.

Variables	ICD-9-CM diagnosis codes	ICD-9-CM procedure codes
Severe sepsis or septic shock	A40, A41, T8144, T8112, R652	---
Acute renal failure requiring dialysis	N17, N990	5A1D70Z, 5A1D80Z, 5A1D90Z
Acute myocardial infarction	I21	---
Pulmonary embolism	I26	---
Cardiac arrest requiring cardiopulmonary resuscitation	I46, I9712	5A12012, 5A1221Z
Prolonged requirement of mechanical ventilation (> 24 hours)	---	5A1945Z, 5A1955Z
Unplanned intubation/reintubation	J95821	0BH13EZ, 0BH17EZ, 0BH18EZ

Table S2. Factors associated with Clavien-Dindo grade IV complications and in-hospital mortality among hospitalizations ≥65 years.

Characteristic	Odds ratio (95% CI)
Year	
2019	Reference
2020	1.27 (0.64-2.50)
Age	1.02 (0.96-1.08)
Sex	
Male	Reference
Female	0.49 (0.24-1.98)
Race	
White	Reference
Black	1.13 (0.87-2.27)
Hispanic	1.70 (0.63-4.57)
Asian or Pacific Islander and Native American	1.83 (0.71-4.70)
Other	2.82 (0.88-3.08)
Insurance	
Medicare	Reference
Medicaid	2.28 (0.54-3.67)
Private insurance	1.19 (0.33-2.29)
Other	1.12 (0.57-1.98)
Hypertension	0.82 (0.37-1.82)
Diabetes mellitus	1.23 (0.86-1.76)
Obesity	1.00 (0.39-2.59)
Coagulation disorder	2.68 (1.00-3.15)
Peripheral vascular disease	0.85 (0.30-2.38)
Liver disease	1.49 (0.30-2.32)
Chronic renal failure	3.45 (1.57-4.60)
Alcohol abuse	1.19 (0.65-1.78)
Drug abuse	1.35 (0.76-2.15)
